# Targeting Actomyosin Contractility Suppresses Malignant Phenotypes of Acute Myeloid Leukemia Cells

**DOI:** 10.3390/ijms21103460

**Published:** 2020-05-14

**Authors:** Fengjiao Chang, So Jung Kong, Lele Wang, Beom K. Choi, Hyewon Lee, Chan Kim, Jin Man Kim, Kyungpyo Park

**Affiliations:** 1 Department of Physiology, School of Dentistry, Seoul National University and Dental Research Institute, Seoul 110-749, Korea; changfengjiao@snu.ac.kr (F.C.); wanglele@snu.ac.kr (L.W.); 2 Medical Oncology, CHA Bundang Medical Center, CHA University, Seongnam 13488, Korea; kongsojung@gmail.com (S.J.K.); chan@cha.ac.kr (C.K.); 3 Laboratory of Translational Immuno-Oncology, Seongnam 13488, Korea; 4 Biomedicine Production Branch, National Cancer Center, Goyang 10408, Korea; 11380@ncc.re.kr; 5 Hematologic Oncology Clinic, Center for Specific Organs Cancer Research Institute & Hospital, National Cancer Center, Goyang 10408, Korea; hwlee@ncc.re.kr; 6 Department of Dentistry, School of Medicine, CHA University, CHA Bundang Medical Center, Seongnam 13488, Korea

**Keywords:** non-muscle myosin II, contractility, malignant phenotypes, acute myeloid leukemia

## Abstract

Actomyosin-mediated contractility is required for the majority of force-driven cellular events such as cell division, adhesion, and migration. Under pathological conditions, the role of actomyosin contractility in malignant phenotypes of various solid tumors has been extensively discussed, but the pathophysiological relevance in hematopoietic malignancies has yet to be elucidated. In this study, we found enhanced actomyosin contractility in diverse acute myeloid leukemia (AML) cell lines represented by highly expressed non-muscle myosin heavy chain A (NMIIA) and increased phosphorylation of the myosin regulatory light chain. Genetic and pharmacological inhibition of actomyosin contractility induced multivalent malignancy- suppressive effects in AML cells. In this context, perturbed actomyosin contractility enhances AML cell apoptosis through cytokinesis failure and aryl hydrocarbon receptor activation. Moreover, leukemic oncogenes were downregulated by the YAP/TAZ-mediated mechanotransduction pathway. Our results provide a theoretical background for targeting actomyosin contractility to suppress the malignancy of AML cells.

## 1. Introduction

A network of actin-myosin cytoskeleton conducts critical and fundamental biological processes [1]. The actomyosin interaction triggers the contractile force that governs the physical separation of daughter cells during cytokinesis [2]. The initiation of cell spreading and migration is also dependent on actomyosin contractile properties [3,4]. In addition, a pivotal role for actomyosin contractility has been proposed in the specific context of bone marrow and blood cells. Actomyosin contractility directs the fate of hematopoietic stem cells (HSCs) [5,6]. Moreover, actomyosin contractility is required in several processes of hematopoietic cells, such as immune cell recruitment, the cytotoxic response of natural killer (NK) cells, and megakaryocyte maturation [7,8,9].

The core mechanism of actomyosin contractility is the coordinated activity of molecular motor myosin II that interacts with its substrate F-actin scaffold. Myosin II molecules consist of three pairs of peptide chains—two heavy chains that include all of the essential elements in their head domains (binding site for both ATP and actin), two regulatory light chains that regulate myosin II activities (ATP hydrolysis and actin-myosin assembly), and two essential light chains [10]. Myosin activity is regulated by the phosphorylation of its regulatory light chains which switches myosin structure from a compact monomeric form to an extended dimeric form and enables the assembling of bipolar myosin filaments. Several upstream proteins, such as Rho GTPases, Rho-associated protein kinase (ROCK), and myosin light chain kinase (MLCK) are known to control myosin activities by transmitting phosphorylation signals on regulatory light chains [11]. The activated myosin filaments slide along antiparallel actin fibers to strengthen actomyosin binding and initiate contraction by the ATPase activity in myosin heads. Myosin ATPase catalyzes ATP into a metastable complex, and the release of the hydrolysis products of ATP transduces chemical energy for direct actin-myosin binding [12].

Actomyosin contractility has crucial roles in biological processes, and therefore its abnormality causes altered diverse cell phenotypes (for example, multinucleated or multipolarized cells, altered motility), which have been critically implicated in numerous pathologies [13]. Notably, the profile of abnormal myosin II activity in tumorigenesis continues to grow, especially in diverse solid tumors. Increased expression or activities of myosin heavy/light chains and their regulators drive tumor invasion and metastasis in breast, pancreatic, melanoma, and glioma cancers [14,15,16,17]. In contrast, reduced myosin functions lead to cytokinesis failure, multinucleation, and multipolar mitosis that enhances genetic instability and malignant transformation [18]. Although multiple studies have pointed out the relevance of actomyosin contractility in malignant phenotypes of various cancer cells, the relevance in hematopoietic malignancy has yet to be clearly identified.

Acute myeloid leukemia (AML) is characterized by the abnormal growth of a clonal population of HSCs and progenitor cells, accounting for about 80% of acute leukemia in adults with an increasing incidence by age [19]. The core event of leukemia progression is the accumulation of immature myeloid progenitor cells, leading to the suppression of normal hematopoiesis in the bone marrow and peripheral blood. However, the biological and clinical significance of actomyosin contractility in leukemogenesis is just beginning to merge. A recent article reported that ROCK hyperactivity was detected in cells bearing leukemogenic mutations, and ROCK inhibitor suppressed the constitutive growth of AML cells in patient samples [20]. Apoptosis and differentiation processes in multiple human leukemia cells are known to be mediated by ROCK and MLCK, respectively [21,22]. Moreover, myosin II was introduced as a key mediator of migration and infiltration into peripheral tissues of acute lymphoblastic leukemia cells [23]. Despite the importance of actomyosin contractility in leukemia being noted, more in-depth studies are required to understand the mechanisms underlying leukemic transformation and to develop an evidence-based strategy for the management of leukemia.

In this study, we evaluated the contribution of actomyosin contractility in regulating the malignant phenotypes of AML cells through multidisciplinary approaches. We found that actomyosin contractility is a critical checkpoint in regulating the multiple parameters of malignant phenotypes of the leukemia cells through distinct signaling pathways. In particular, perturbing actomyosin contractility by targeting myosin ATPase showed versatile effects on the alleviation of leukemic cell malignancies. These results could provide a theoretical background for a potential therapeutic approach for AML.

## 2. Results

### 2.1. Hypercontractility of Actomyosin Complex in AML Cells

**Figure 1:**
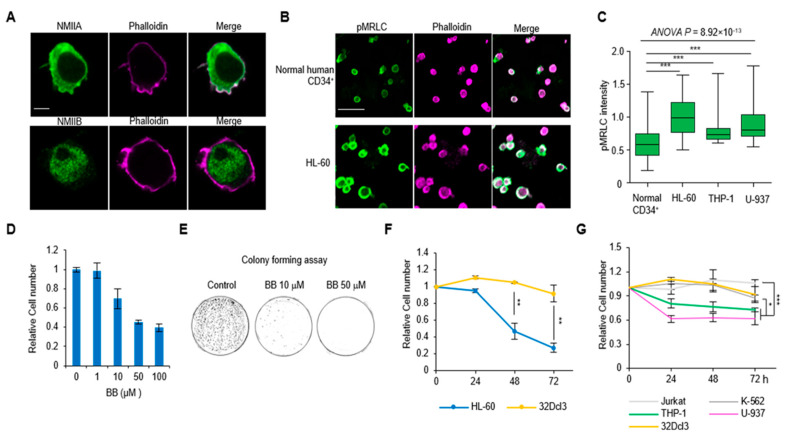
The relationship of actomyosin contractility and acute myeloid leukemia (AML) cell growth. (**A**) The localization of non-muscle myosin II (NMII) A or B (green) and their spatial relationship with phallodin (magenta) in AML cell line HL-60. (**B**) Immunofluorescence images of the phosphorylation level of the myosin regulatory light chain (pMRLC) expression between normal CD34^+^ cells and HL-60 cells. (**C**) Quantification of the expression of pMRLC in AML cell lines (THP-1 and U-937) (CD34^+^: *n* = 67; HL-60: *n* = 44; THP-1: *n* = 39; U-937: *n* = 71). Data are presented as median ± min/max. (**D**) Viable HL-60 cells counted after treatment with the indicated dose of blebbistatin (BB) in 24 h (*n* = 3). Data are represented as mean ± SEM. (**E**) Representative images of the colonies of HL-60 cells in methylcellulose-based medium with blebbistatin treatment. (**F**) The results of blebbistatin (50 µM) induced cell number changes between normal 32Dcl3 myeloid cells and HL-60 cells in a time-dependent manner (*n* = 6). Data are represented as mean ± SEM. (**G**) Quantification of the cell number changes of various leukemic cell lines upon 50 µM blebbistatin treatment (*n* = 6). Data are represented as mean ± SEM. Scale bars: 5 µm (A), 50 µm (B). * *p* < 0.05, ** *p* < 0.01, *** *p* < 0.001.

We first investigated the expression patterns of non-muscle myosin II (NMII) isoforms and their spatial relationship with filamentous actin (F-actin) in AML cells. Hematopoietic lineages differentially express two heavy chain isoforms of NMIIA and NMIIB. In human leukemia (HL)-60 cells, a representative acute promyelocytic leukemia cell line [24], NMIIA and NMIIB have distinct cellular localization patterns (Figure 1A). NMIIAs are primarily distributed on the plasma membrane and the rear region (opposite the protrusive area) of the cell with lower cytoplasmic expression. However, NMIIBs are localized in the nucleus and cytoplasm (Figure 1A and Figure S1A). Notably, the co-localization of NMIIA on plasma membrane and cortical actin was clearly exhibited (Figure 1A). The BloodSpot database (www.bloodspot.eu) reported that NMIIA mRNA expression was increased in primary human AML across cytogenetic subgroups relative to normal HSCs and progenitor controls [25] (Figure S1B). However, BloodSpot analysis showed no significant elevation of NMIIB expression in AML subsets (Figure S1C). We next investigated the phosphorylation level of the myosin regulatory light chain (pMRLC) in AML cells. We found that pMRLC levels are significantly higher in all the AML cell lines (HL-60, THP-1, U-937) investigated compare to normal human CD34^+^ cells (*p* < 0.001; Figure 1B,C and Figure S1D). These data indicate that AML cells have a highly contractile phenotype which is mediated by the NMIIA-actin network with increased pMRLC levels.

### 2.2. Perturbation of Actomyosin Contractility Suppresses the Growth of AML Cells

We next evaluated the effects of blebbistatin treatment on actomyosin contractility in AML cells. Blebbistatin is a reversible inhibitor of myosin Ⅱ ATPase, which binds to a cleft between the actin and ATP binding regions and inhibits inorganic phosphate (Pi) release in the MgADP-Pi complex, resulting in the detachment of actin and myosin head [26]. Blebbistatin treatment decreased HL-60 cell numbers in a dose-dependent manner (Figure 1D). In long-term culture (14 days) with methylcellulose-based medium, the colony formation of HL-60 cells was markedly and dose-dependently diminished in blebbistatin-treated groups (Figure 1E). We next compared the effect of blebbistatin treatment on the changes of cell numbers in 32D Clone 3 (32Dcl3) cells, a nontumorigenic myeloid cell line [27], and HL-60 cells. HL-60 cells showed a significant reduction of cell number (48 h: 53.4%; 72 h: 72.82%), whereas there was only 8.15% reduction with no significance in 32Dcl3 cells at 72 h (Figure 1F). In addition, the effects of blebbistatin on other type of leukemic cells were explored, including Jurkat cells (acute lymphoblastic leukemia), K-562 cells (chronic myeloid leukemia), and other AML cells (THP-1 and U-937). It is noteworthy that both THP-1 and U-937 cells responded more sensitively to blebbistatin than Jurkat and K-562 cells (Figure 1G), indicating that blebbistatin has a specific effect on AML cell types.

### 2.3. Perturbation of Actomyosin Contractility Enhances Apoptosis of AML Cells

**Figure 2:**
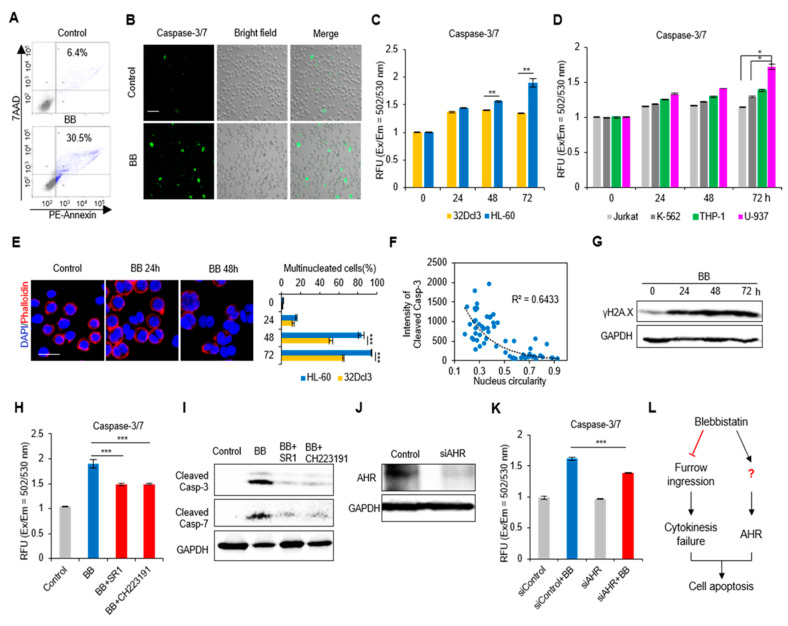
Perturbation of actomyosin contractility effects apoptosis of AML cells. (**A**) Flow Cytometry analysis of cellular apoptosis after 24 h blebbistatin (BB, 50 µM) treatment. Annexin V^+^ cells are highlighted in blue color. (**B**) Immunofluorescence images of caspase-3/7 activity upon 50 µM blebbistatin treatment in HL-60 cells. (**C**) Quantification of caspase 3/7 apoptosis signal of 32Dcl3 and HL-60 cells after 50 µM blebbistatin treatment in a time-dependent manner. Data are measured as the fluorescence (Excitation / Emission = 502/530 nm) (*n* = 3). Data are represented as mean ± SEM. (**D**) Comparison of apoptosis level in Jurkat, K-562 with other AML cell lines (*n* = 6). Data are represented as mean ± SEM. (**E**) Left panel: representative images of cell multinucleation with blebbistatin treatment in HL-60 cells. Right panel: quantification of changes in multinucleation of 32Dcl3 and HL-60 cells over time (*n* = 3). Data are represented as mean ± SEM. (**F**) The correlation of cleaved caspase-3 expression with cell nucleus circularity (*n* = 62). (**G**) Immunoblotting of γH2A.X upon blebbistatin treatment in a time-dependent manner in HL-60 cells. (**H**) The effects of AHR antagonists (SR1 and CH223191) on blebbistatin induced HL-60 cell apoptosis (*n* = 6). Data are represented as mean ± SEM. (**I**) The effects of AHR antagonists on blebbistatin activated the caspase-3/7 apoptotic pathways. (**J**) The silencing efficiency of AHR siRNA. (**K**) The genetic perturbation of AHR affects blebbistatin induced HL-60 cell apoptosis (*n* = 6). Data are represented as mean ± SEM. (**L**) Experimental scheme of signaling nodes in blebbistatin induced HL-60 cell apoptosis. Scale bars: 50 µm (B), 20 µm (E). * *p* < 0.05, ** *p* < 0.01, *** *p* < 0.001.

We next investigated the mechanism of the blebbistatin-induced decrease in cell number. First, we found that there was a remarkable increase of apoptosis in HL-60 cells upon 24 h blebbistatin treatment [Annexin V^+^ cells: 6.4% (Control) versus 30.5% (Blebbistatin); Figure 2A]. HL-60 cells also showed enhanced caspase 3/7 apoptotic signal in the presence of blebbistatin (Figure 2B). The caspase-3/7 apoptosis signal of 32Dcl3 cells was increased to a similar extent of that observed in HL-60 at 24 h (40.72 ± 3.92% (32Dcl3) versus 44.53 ± 3.37% (HL-60); *p* = 0.42; Figure 2C) and sustained an apoptotic level until 72 h. However, HL-60 cells rapidly experienced an increase in apoptosis demonstrated by strongly enhanced caspase-3/7 signals (90.17 ± 0.08% increase at 72 h). Furthermore, the apoptotic effects of blebbistatin on other leukemia cell lines showed that AML cell lines presented higher apoptotic tendency upon blebbistatin treatment (Figure 2D). Next, we genetically perturb actomyosin contractility by generating a HL-60 cell line that stably expresses non-phosphorylatable MLC mutant (T18A/S19A) tagged with EGFP (MRLC-AA-EGFP) and evaluated cell viability. The mutant cells showed stable expression of EGFP signals and markedly decreased pMLC level (Figure S2A,B). As expected, there were decreased cell viability in MRLC-AA expressing cells in comparison with control EGFP expressing HL-60 cells (Figure S2C–E).

Blebbistatin treatment arrests furrow ingression during cytokinesis, the production of multinucleated cells that undergoes diverse fates, including cell death by apoptosis [9]. Consistent with this previous report, we observed that HL-60 cells exhibited a strikingly increased rate of multinucleated cells during 72 h blebbistatin treatment (24 h: 15.85 ± 0.76 %, 48 h: 83.46 ± 3.29%, 72 h: 94.24 ± 0.55%; Figure 2E), which was significantly higher than 32Dcl3 cells (Figure 2E). The circularity of the nucleus tends to decrease in the presence of an abnormal number or status of chromosomes. An inverse correlation of nucleus circularity and cleaved caspase-3 apoptotic signals was observed (R^2^ = 0.6433; Figure 2F), and the actual chromosomal damage was confirmed by an increase in the phosphorylation of histone H2A.X (γH2A.X) upon blebbistatin treatment (Figure 2G). These results indicate that blebbistatin-induced chromosomal abnormality can be a direct mechanism underlying the apoptotic process of HL-60 cells.

The aryl hydrocarbon receptor (AHR) is a ligand-induced transcription factor that influences the major stages of tumorigenesis and is also known to be a key signaling mediator in blebbistatin induced-apoptosis of hematopoietic cells [6]. We hypothesized that there is an association between AHR and the induction of the apoptosis in AML cells. As expected, both AHR antagonists partially but significantly rescued blebbistatin-induced caspase 3/7 activation (StemRegenin-1 (SR1): *p* = 1.69 × 10^−4^; CH223191: *p* = 5.70 × 10^−5^; Figure 2H). Moreover, the increased amount of cleaved caspase 3/7 by blebbistatin treatment was reduced by both antagonists (Figure 2I). Inhibition of AHR using RNA interference (RNAi)-mediated silencing also rescued blebbistatin-induced caspase activation (*p* = 1.95 × 10^−5^; Figure 2J,K). Taken together, these findings reveal that blebbistatin-enhanced cell apoptosis is mediated by cytokinesis failure and by the AHR-caspase signaling pathway (Figure 2L).

We also found a significant reduction of cell mobility upon blebbistatin treatment. The transmigration of HL-60 cells was decreased upon blebbistatin (1.76 ± 0.08% (control) versus 0.65 ± 0.14% (blebbistatin); Figure S3A,B), and the two-dimensional cell trajectories highlighted that blebbistatin-treated cells displayed a shorter migration path, indicating blocked intrinsic motility (migration speed: 285.09 ± 7.29 µm/h (control) versus 92.02 ± 3.42 µm/h (blebbistatin); Figure S3C,D). These results demonstrate that blebbistatin has a multivalent effect in the regulating diverse functions of AML cells, such as survival and mobilization.

### 2.4. Actomyosin Contractility Regulates Transcriptional Activities through the YAP/TAZ Pathway

**Figure 3:**
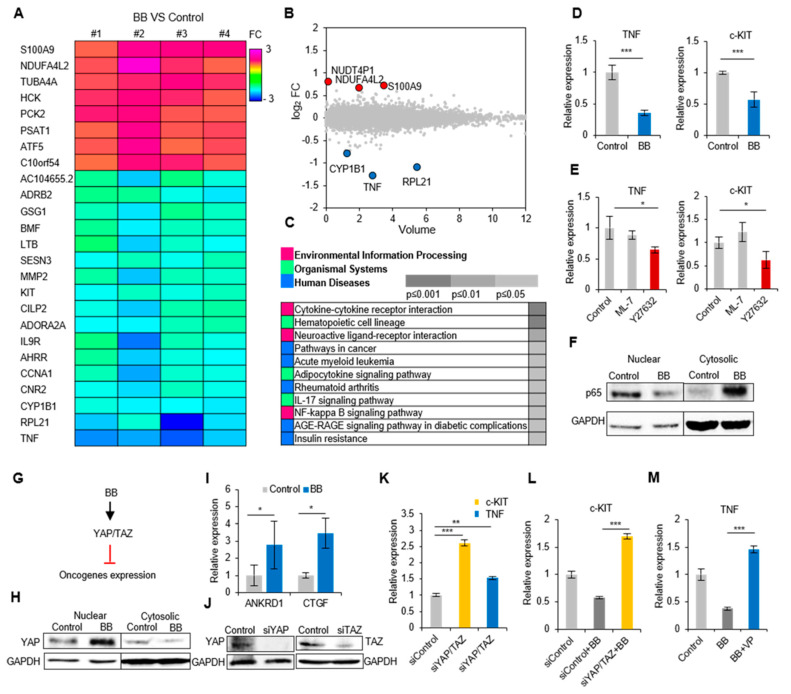
Perturbation of actomyosin contractility induces transcriptional changes in AML cells. (**A**) Heat map of significantly up and downregulated genes (fold change ≥1.5) by 50 μM blebbistatin in HL-60 cells (*n* = 4). (**B**) The top six genes are shown in a volume plot. Red dots represent upregulated genes, and blue dots represent downregulated genes. (**C**) KEGG pathway analysis of blebbistatin modulated enriched pathway in HL-60 cells. (**D**) Blebbistatin-induced oncogenes changes were verified by qRT-PCR (*n* = 6). Data are represented as mean ± SEM. (**E**) Myosin activity induced oncogenes changes in HL-60 cells (*n* = 3). ML-7: MLCK inhibitor, Y27632: ROCK inhibitor. Data are represented as mean ± SEM. (**F**) The involvement of NF-κB signaling was confirmed by immunoblotting. (**G**) Experimental design of signaling nodes in blebbistatin (BB) induced oncogenes changes. (**H**) Immunoblotting of YAP activation upon blebbistatin treatment. (**I**) The expression of YAP regulated genes (*ANKRD1*, *CTGF*) were verified using qRT-PCR (*n* = 3). Data are represented as mean ± SEM. (**J**) The silencing efficiency of YAP/TAZ siRNA. (**K**) mRNA expression of c-KIT and TNF in Yes-associated protein (YAP)/ transcription regulator protein 1 (TAZ) double knock down HL-60 cells (*n* = 3). Data are represented as mean ± SEM. (**L**) The siYAP/TAZ affects blebbistatin induced *c-KIT* downregulation. Data are represented as mean ± SEM. (M) The verteporfin (YAP–TEAD binding inhibitor) affects blebbistatin induced *TNF* downregulation (*n* = 3). Data are represented as mean ± SEM. * *p* < 0.05, ** *p* < 0.01, *** *p* < 0.001.

To gain a better understanding of blebbistatin-induced transcriptional changes of AML cells, the gene expression profiles of HL-60 cells were analyzed by whole transcriptome analysis using RNA sequencing (RNA-seq). In blebbistatin-treated cells, there were 25 differentially expressed genes (DEGs) that were not differentially expressed in non-treated cells (≥1.5-folds, *p* < 0.05; Figure 3A). We listed the six top-ranked genes in a volume plot (three upregulated and three downregulated; Figure 3B), and KEGG pathway enrichment analysis was performed to identify the enriched pathways (*p* < 0.05) in the gene sets (Figure 3C). Among the DEG list, we identified the downregulation of several genes known to be highly related to hematopoietic malignancies, *TNF* [28,29], *c-KIT* [30], and *MMP-2* [31]. Downregulation of identified oncogenes (*TNF* and *c-KIT*) were confirmed by quantitative real-time polymerase chain reaction (qRT-PCR) (Figure 3D). The downregulation of *TNF* and *c-KIT* in other AML cell lines were also detected upon blebbistatin treatment, but HL-60 cells responded the most sensitively (Figure S4A,B). We also revealed that ROCK, but not MLCK, is an upstream mediator of these transcriptional changes by using treatment with inhibitors of both kinases (Y27632 and ML-7) (Figure 3E). Additionally, decreased TNF expression upon blebbistatin treatment was verified by the nucleus-to-cytoplasm translocation of p65, indicating downregulation of TNF-NFκB signaling activity [32] (Figure 3F).

We next focused on the molecular mechanism underlying blebbistatin-induced transcriptional changes. Recent studies have demonstrated that cell contractile forces are able to regulate gene expression in association with Yes-associated protein (YAP) and WW-domain-containing transcription regulator protein 1 (WWTR1 or TAZ) [33]. We thus hypothesized that the genetic changes upon blebbistatin treatment were mediated by YAP/TAZ activity in AML cells (Figure 3G). We observed the YAP translocation from the cytoplasm to nucleus and YAP related genes (*ANKRD1*, *CTGF*) also upregulated upon blebbistatin treatment, indicating that blebbistatin enhanced YAP/TAZ activity (Figure 3H,I). Inhibition of YAP and TAZ using RNAi-mediated silencing enhanced *c-KIT* and *TNF* expression (Figure 3J,K), an opposite effect to that found after blebbistatin treatment (Figure 3D). In addition, YAP/TAZ double knockdown and verteporfin (YAP-TEAD binding inhibitor) treatment rescued the blebbistatin suppressed *c-KIT* and *TNF* expression, respectively (Figure 3L,M). These data demonstrate that YAP/TAZ is a critical mediator in transcriptional changes of HL-60 cells upon blebbistatin treatment.

### 2.5. Blebbistatin Treatment Suppresses Leukemia Progression with Enhanced Apoptosis

**Figure 4:**
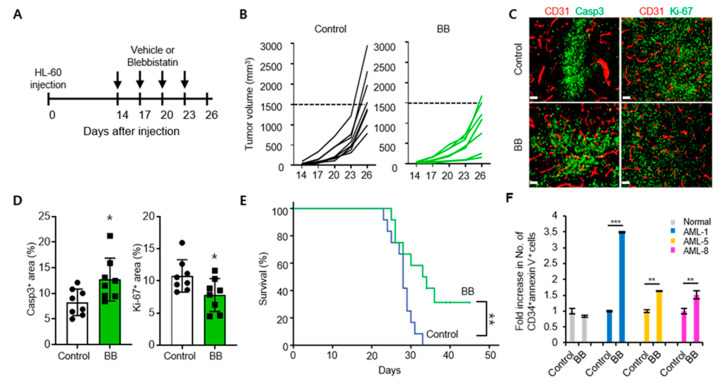
Blebbistatin treatment delays leukemia progression through enhanced apoptosis. (**A**) Mice were subcutaneously implanted with 2 × 10^6^ HL-60 cells and treated with intraperitoneal injections of control (vehicle) or blebbistatin (BB, 2 mg/kg). Diagram depicting treatment schedule. Arrows indicate treatment. (**B**) Comparison of HL-60 tumor growth (*n* = 8 per group). (**C**,**D**) Representative images (**C**) and comparisons (**D**) of Caspase-3^+^ apoptotic cells and Ki-67^+^ proliferating cells within tumors (*n* = 8 per group). Values are mean ± SD. (**E**) Kaplan–Meier survival curves for overall survival (*n* = 12 per group). (**F**) Flow Cytometry analysis of cellular apoptosis (plotted as fold change relative to baseline) in PBMCs from AML patients (*n* = 3) and healthy donors (*n* = 2) 72 h after treatment with 50 μM blebbistatin. Data shown as mean ± SEM. Scale bars, 100 μm (C). * *p* < 0.05, ** *p* < 0.01, *** *p* < 0.001.

To confirm the in vivo anti-tumor efficacy of blebbistatin on leukemia, NOD-SCID mice were implanted with HL-60 cells and treated with either control (vehicle) or blebbistatin at the given time-points (Figure 4A). Repeated treatments of blebbinstatin treatment suppressed the growth of HL-60 leukemia by 42% when compared to the control tumors (Figure 4B). Histologic analyses showed a 1.5-fold increase in the Caspase-3^+^ apoptotic area and 28% decrease in Ki-67^+^ proliferating cells in blebbistatin-treated tumors (Figure 4C,D), which are consistent with in vitro findings of this study. The survival of blebbistatin-treated mice was longer than that of the control mice (Figure 4E). Finally, we verified the anti-leukemic effect of blebbistatin in human AML patient samples. The cells derived from AML patients showed an apoptotic response after 72 h blebbistatin treatment (analyzed by CD34^+^ and annexin V double staining), whereas samples from normal donors showed low sensitivity to blebbistatin (Figure 4F). Collectively, blebbistatin treatment was found to effectively inhibit leukemia progression through the enhanced apoptosis of tumor cells both in a xenograft mice model and in AML patient-derived cells.

## 3. Discussion

In this study, we found that in AML cells, NMIIAs were highly expressed and colocalized with cortical actin scaffolds, which are essential components of the actomyosin contractile complex. In addition, the phosphorylation of the myosin regulatory light chain was also increased, indicating the hypercontractility of AML cells relative to normal controls. A number of studies have revealed that the differential expression and/or activation of myosin II, MLCK, Rho GTPases, and ROCK, commonly indicate enhanced actomyosin contractility and promotes cancer progression across a variety of solid tumors [13,34,35]. The contractility of tumor cells is generated mainly by actin stress fibers derived from cell-matrix adhesion whereas cortical actin patterns with low adhesion level are represented in blood cell types [36]. The structurally different cytoskeleton implies the possibility of a unique regulatory mechanism and drug response in leukemia cells distant from those in adherent tumor cells. However, the relevance of actomyosin contractility in the field of hematopoietic cells is only recently beginning to be elucidated. The Bloodspot analyses showed increased NMIIA and decreased NMIIB levels in AML samples compared to normal HSCs and myeloid progenitors (Figure S1B,C). Overexpressed NMIIA associates with shorter overall survival in patients with different types of cancer, including AML [37,38]. NMIIB polarizes near a cleavage furrow and physically break the symmetry of cytokinesis into asymmetric division [39]. It is therefore predictable that NMIIB correlates with a half-dozen genes involved in the differentiation of hematopoietic cells [40]. Reduced NMIIB indicates the suppression of normal hematopoiesis in the bone marrow and peripheral blood in AML, which also has remarkable clinical significance. Other studies have reported the critical roles of actomyosin contractility in the self-renewal and differentiation process of HSC and progenitor cells [41,42]. This background inspired us to investigate the contribution of actomyosin contractility in regulating malignant phenotypes of AML cells. Pharmacological and genetic perturbation of actomyosin contractility showed multivalent malignancy-suppressive effects on various cellular parameters such as proliferation, survival, and migration. Moreover, transcriptome analysis discovered a decrease in the expression of leukemic oncogenes and enriched AML related pathways. The previous reports and our results commonly highlight that more attention is needed to dissect the mechanism associated with actomyosin machinery in leukemia progression.

We observed that blebbistatin treatment elicited the amplification of bi- or multinucleated (polyploid) cells. Notably, this event was highly correlated with the sustained increase of apoptosis. Actomyosin contractility triggers a driving force to ingress the cleavage furrow in cytokinesis. When blebbistatin was treated, the proliferating cells failed to abscise and to segregate chromosomes [43]. The resultant genomic instability induces a P53-dependent cell cycle arrest, known as the G1-tetraploidy checkpoint, which is a principal process in tumor suppression [44]. Our data demonstrate that this checkpoint system is a core mechanism in blebbistatin-induced apoptosis of AML cells. We also found a higher apoptotic rate in AML cells compared to normal hematopoietic cells upon blebbistatin treatment. The different reactions might be due to two factors. First, the hypercontractility observed specifically in AML cells (Figure 1A,B) can be a reason for the high sensitivity to the reaction of blebbistatin. Second, the high proliferation rate in AML cells increases the chance of cell cycle arrest on blebbistatin treatment, in which the accumulation of genomic damages is rapidly increased (Figure 2G). 

In addition, we revealed AHR-induced caspase activation by blebbistatin treatment and suggest it as a supportive signaling in the apoptotic process. Together with the results of rescue experiments of AHR inhibition (Figure 2H–K), RNA-seq results discovered that the expression of the aryl hydrocarbon receptor repressor (AHRR) decreased (Figure 3A), supporting the evidence that blebbistatin treatment activates the AHR pathway. Meanwhile, a previous study suggested that blebbistatin could directly activate AHR because of its structural analogy with AHR ligands [6]. Therefore, the exact mechanism of actomyosin contractility in regulating AHR signaling activity remains to be explored.

Increasing evidence has been concerned with the crucial prognostic value of genetic abnormalities in AML patients, especially in whether the presence of activated oncogenes is likely to trigger hematopoietic malignancies [45]. Based on RNA-seq profiles, we identified the downregulation of leukemic oncogenes, KIT, and TNF upon blebbistatin treatment. KIT belongs to the class III receptor tyrosine kinases (RTK) and is one of the most frequently disordered genes in AML [30]. Recent studies showed that increased phosphorylation of the myosin light chain promotes cytoskeletal contractility in the KIT-bearing AML cells leading to cell growth and survival [20,46]. Elevated TNF levels are primarily responsible for the constitutively activated NF-κB, which has been reported to stimulate the growth of AML cells and repress normal HSPCs growth [32,47]. We anticipate that contractility-mediated oncogene regulation can be a crucial checkpoint for determining the prognosis of diverse types of hematologic malignancy.

Thus, how does actomyosin contractility regulate gene expression in AML cells? We revealed that the key mediators that link the membrane-to-nucleus process are YAP and TAZ, which are representative mechanotransducers that translate mechanical cues into biochemical signals [33]. It has been reported that high contractility of the cells elicits nuclear transport of YAP and TAZ, resulting in the interaction with transcription factors of the TEA domain (TEAD) family members to regulate gene expression [33]. However, our results showed that inhibition of actomyosin contractility induces YAP nucleus translocation (Figure 3H), which is a conflicting result to the generally accepted concept. Several studies indicated that actomyosin-based mechanical forces have different effects on YAP and TAZ localization depending on the cellular context [48,49]. In line with our finding, the inhibition of actomyosin contractility in epithelial monolayer facilitates the nuclear localization of YAP [48]. Interestingly, the epithelial monolayer cells presented well-organized cortical actin cables reminiscent of the actin patterns of AML cells observed in this study. Moreover, YAP1 activation triggers apoptosis specifically in hematological malignancies, including lymphomas, leukemias, and multiple myeloma [50,51]. Therefore, the context-dependent YAP/TAZ regulatory mechanism might be mainly due to the structural characteristics of the actomyosin network.

**Figure 5:**
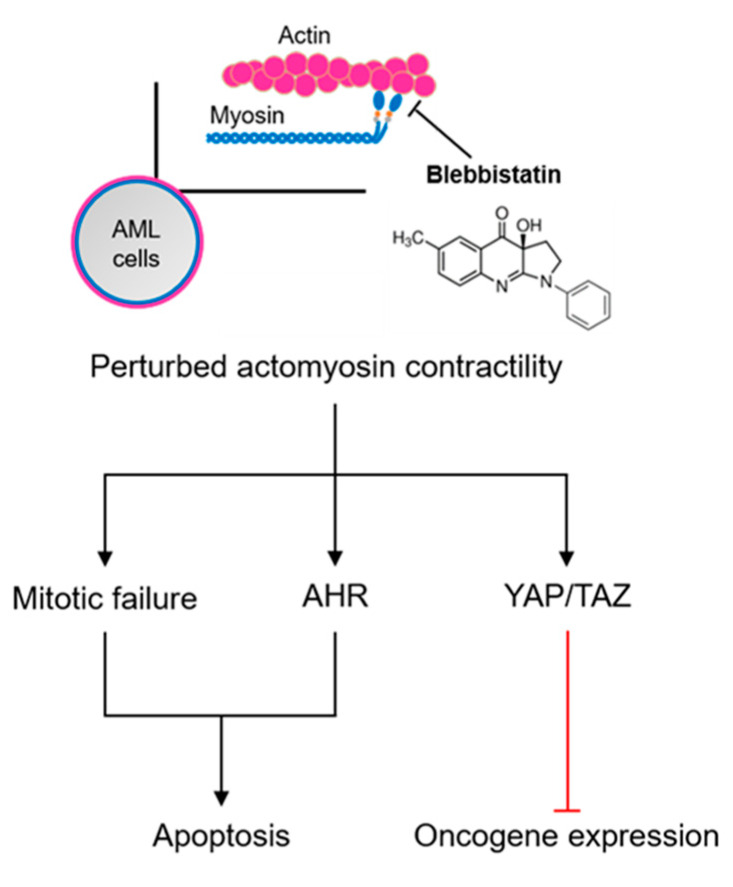
Schematic representation of actomyosin contractility-mediated AML progression. Perturbation of actomyosin contractility by myosin ATPase inhibition (blebbistatin) induces AML cell apoptosis and oncogene downregulation. Mitotic failure and AHR activation contribute to the apoptotic process, and YAP/TAZ transcriptional activity leads to the downregulation of leukemic oncogenes.

Throughout this study, we demonstrated that actomyosin contractility could be a critical target for an AML therapeutic strategy. The restrained AML progression by the loss of actomyosin contractility is manifested by a proliferative and survival disadvantage, and the transcriptome analysis reveals the downregulation of leukemic oncogenes and activated pathways (Figure 5). While the global inhibition of the actin cytoskeleton would have extensive adverse effects, blebbistatin functions as a specific myosin II ATPase inhibitor that has been developed to evaluate the role of cytoskeletal activities under a variety of biological conditions. The binding of blebbistatin to myosin II heads could directly suppress terminal actin-myosin attachment and reduce the broad side effects instead of their upstream regulators. However, the application of blebbistatin has several limitations, such as low solubility, cytotoxicity, and structural instability [52]. Further exploration of highly potent and biological safe blebbistatin derivatives [53] would be critical to implement AML therapy that targets actomyosin activity.

## 4. Materials and Methods

### 4.1. Antibodies and Reagents

Rabbit purified non-muscle myosin heavy chain II-A (909801) and non-muscle myosin heavy chain II-B antibodies (909901) were purchased from BioLegend (San Diego, CA, USA). Rabbit phospho-myosin light chain 2 (ser19) (3671), cleaved caspase-3 (Asp175) (5A1E) (9664), caspase-7 (9492), NF-κB p65 (C22B4) (4764), YAP (D8H1X) XP (14074) and TAZ (D3I6D) (70148), and Ki-67 antibodies (9027) were purchased from Cell Signaling Technology (Danvers, MA, USA). Alexa Fluor 488 Anti-Human CD11b (561687), Alexa Fluor 488 Anti-Human CD14 (561706), PE Annexin V (559763), and CD34 APC antibodies (345804) were purchased from BD Biosciences (San Jose, CA, USA). Mouse aryl hydrocarbon receptor (AHR) antibody (sc-133088) was purchased from Santa Cruz Biotechology (Santa Cruz, CA, USA). Hamster anti-CD31 antibody (MAB1398Z) was purchased from Millipore (Oakville, ON, Canada). Rabbit anti-Caspase-3 antibody (AF835) was purchased from R&D Systems (Minneapolis, MN, USA).

Blebbistatin (B0560), ML-7 (I2764) and Verteporfin (SML0534) were purchased from Sigma Aldrich (St. Louis, MO, USA). StemRegenin 1 (SR1) (C7710-1) was purchased from Cellagen Technology (San Diego, CA, USA). CH229131 (3858) and Y27632 (1254), were purchased from Tocris Bioscience (Minneapolis, MN, USA).

### 4.2. Cell Culture and Human Patient Samples

The human leukemia cell lines HL-60 (10240), THP-1 (40202), U-937 (21593.1), Jurkat (clone E6-1, 40152), and K-562 (10243) were purchased from the Korean cell line bank (Seoul, Korea). The murine myeloid 32D Clone 3 (32Dcl3) cell line was purchased from ATCC (CRL-11346, Manassas, VA, USA). All cells were cultured in RPMI-1640 (SH30027.01, Hyclone, Logan, UT, USA) supplemented with 10% FBS (SH30084.03, Hyclone, Logan, UT, USA), 1% sodium pyruvate (11360-070, Gibco, Thermo Fisher Scientific, Waltham, MA, USA), and 1% penicillin/streptomycin (15140-122, Gibco, Thermo Fisher Scientific, Waltham, MA, USA) at 37 °C with 5% CO_2_. 32Dcl3 cells were supplemented with 10 ng/mL murine IL-3 (213-13, Peprotech, Rocky Hill, NJ, USA). The stable cell lines HL-60-EGFP and HL-60 mMYL9-EGFP (MRLC-AA) were obtained from Sirion Biotechnology (Martinsried, Germany) and cultured in medium with 0.5 µg/mL puromycin to select the transduced cells. Analysis of blood samples of AML patients was approved by the Institutional Review Board of the National Cancer Center (Goyang, Korea, NCC2019-0029) in accordance with relevant guidelines and regulations. All blood samples were obtained after informed consent. Patient characteristics are listed in Table S1. Frozen normal peripheral blood mononuclear cells (PBMC) were purchased from stem cell technologies (70025.2, Vancouver, BC, Canada). Primary CD34^+^ cells were sorted from normal mononuclear cells of peripheral blood using a BD-Aria III instrument (San Jose, CA, USA). Primary cells were grown in RPMI-1640 medium supplemented with 20% FBS 1 day prior to the indicated treatment.

### 4.3. Cell Number Count

Cell number of various myeloid cells was assessed after 24, 48, and 72 h in the presence of various concentrations (1–100 μM) of blebbistatin. Viable cell numbers were determined by using trypan blue dye exclusion.

### 4.4. Colony Forming Assay

HL-60 cells (2000 cells/well) were seeded in MethoCult H4230 (Stem cell Technologies, Vancouver, BC, Canada) with 10–50 µM blebbistatin at 37 °C with 5% CO_2_. Colonies were counted after 14 days. Images were captured using a gel documentation imaging system (Bio-Print, Vilber, Eberhardzell, France).

### 4.5. Immunofluorescence

HL-60, THP-1, U-937, Jurkat, K-562 cells, and 32Dcl3 cells (1 × 10^5^ cells/well) were plated on a 96-well glass bottom plate (0611129L2L, Matrical Bioscience, Spokane, WA, USA) coated with Cell-Tak (354240, Corning, Corning city, NY, USA). The specimens were washed, fixed and permeabilized. Next, blocking solution which included 10% FBS and 0.1% BSA were added for 1 h at room temperature. Cells were then incubated overnight at 4 °C with anti-non-muscle myosin heavy chain II-A and B, phospho-myosin light chain 2 (Ser19) and cleaved caspase-3 antibodies (1:200) followed by Alexa Fluor 488 goat anti-rabbit IgG antibodies (1:500; A11034, Invitrogen, Waltham, MA, USA) for 1 h. DAPI (Vector Laboratories, Burlingame, CA, USA) and phalloidin (T7471, Invitrogen, Waltham, MA, USA) were used as a nuclear and cytoskeleton stain. Images were captured on a laser scanning confocal microscope (LSM 700, Carl Zeiss, Oberkochen, Germany).

### 4.6. Western Blotting

HL-60 whole cell lysates were collected in CRB buffer (FNN0011, Invitrogen, Waltham, MA, USA) supplemented with a protease and phosphatase inhibitor cocktail (78443, Thermo Scientific, Waltham, MA, USA). HL-60 cells nuclear and cytoplasmic separation was performed by nuclear and cytoplasmic extraction reagents (78833, Pierce, Waltham, MA, USA). Protein samples were fractionated by SDS-PAGE and transferred to nitrocellulose membranes using an iBLOT 2 Dry Blotting system (IB21001, Thermo Scientific, Waltham, MA, USA), The membranes were then blocked and incubated with phospho-myosin light chain 2 (Ser19), cleaved capspase-3, caspase-7, AHR, YAP, TAZ, and NF-κB p65 at 1:500 concentrations. Immunoreactive bands were ultimately visualized using ECL reagents (32106, Thermo Scientific, Waltham, MA, USA) and detected with Chemidoc XRS+ system (Bio-Rad Laboratories, Hercules, CA, USA).

### 4.7. Caspase-3/7 Apoptosis Test

Cell apoptosis was detected by caspase 3/7 Green Detection Reagent (C10423, Invitrogen, Waltham, MA, USA). Cells were plated in black 96-well plates at a density of 1 × 10^5^ cells per well and cultured in complete medium with 50 μM blebbistatin. They were then treated with 2 μM caspase 3/7 regent for 30 min. The fluorescence (excitation/emission = 502/530 nm) was measured to determine cell activity in triplicate wells.

### 4.8. Flow Cytometry Analysis

Cell apoptosis were checked by flow cytometry. HL-60 cells were washed twice with PBS then stained with phycoerythrin (PE)-conjugated annexin V and 7-amino-actinomycin (7-AAD) for 15 min at room temperature to assess apoptosis, HL-60 EGFP and HL-60 MRLC-AA-EGFP were stained with propidium iodide solution (PI, P4864, Sigma Aldrich, St. Louis, MO, USA) just prior to apoptosis analysis. Total PBMCs were stained with stem and progenitor cell surface markers CD34^+^ and PE annexin V at room temperature for 30 min in the dark. Stained cells were transferred to 5 mL FACS tubes and analyzed by flow cytometry using a BD-Aria III instrument (BD Biosciences, San Jose, CA, USA).

### 4.9. Real-Time PCR and Transcriptome Sequencing

Quantitative real-time PCR was performed using cDNA and SYBR PCR master mix (4309155, Applied BioSystems, Waltham, MA, USA). Samples were analyzed with an Applied Biosystems 7300 instrument. The following primers were used: *TNF*: forward 5′-AGACGCCACATCCCCTGACAA-3′, and reverse 5′-GACGGCGATGCGGCTGATG-3′; *KIT*: forward 5′-TCATGGTCGGATCACAAAGA-3′, and reverse 5′-AGGGGCTGCTTCCTAAAGAG-3′; *ANKRD1*: forward 5′-AGC CCA GAT CGA ATT CCG TG-3′, and reverse 5′-CTC CTT CTC TGT CTT TGG CGT-3′; *CTGF*: forward, 5′-ACCGACTGGAAGACACGTTTG-3′, and reverse 5′-CCAGGTCAGCTTCGCAAGG-3′; *GAPDH*: forward 5′-CAA AGT TGT CAT GGA TGA CC-3′, and reverse 5′-CCA TGG AGA AGG CTG GGG-3′. For transcriptome sequencing, RNA was isolated from HL-60 cells after 16 h of treatment with 50 µM blebbistatin or the vehicle. The mRNA-Seq sample was obtained using an Illumina TruSeq™ RNA Sample Preparation Kit (San Diego, CA, USA). Gene expression data preprocessing and normalization were performed using the fragments per kilobase of exon per million fragments mapped (FPKM) method. Analysis using the Illumina platform was performed at Macrogen.

### 4.10. Small Interfering RNA

To transiently knock down the expression of AHR, YAP, and TAZ, HL-60 cells were transfected with ON-TARGETplus SMARTpool siRNAs (Dharmacon, Cambridge, UK) via electroporation using the Neon transfection system (MPK5000, Invitrogen). The following siRNA sequences were used: AHR: GCAAGUUAAUGGCAUGUUU, GAACUCAAGCUGUAUGGUA, GCACGAGAGGCUCAGGUUA, GCAACAAGAUGAGUCUAUU. YAP1: CCGAAAUCUUGGACGUGGA, GAAUAAAGGAUGGCGUCUU, UCUUAAAUCACAACGAUCA, AAGGAGAGACUGCGGUUGA. TAZ1: CAAUUUAUGUCCACGUUAA, CCAUUGAAAUAGAAACGCA, GAGAUGACCUUCACGGCCA, AUGUAUUGGCAGACGAGAA. The nontargeting siRNA sequences were: UGGUUUACAUGUCGACUAA, UGGUUUACAUGUUGUGUGA, UGGUUUACAUGUUUUCUGA, UGGUUUACAUGUUUUCCUA. siRNAs were used at a final amount of 100 nM. Knockdown efficiency was determined by western blotting 48 h after transfection.

### 4.11. Cell Migration Test

For Transwell assays, HL-60 cells (2 × 10^5^) in serum-free medium were added to the upper chamber of a 5 μm pore size Transwell membrane filter (3421, Corning, Corning city, NY, USA) and 10% FBS was added to the bottom chamber for use as a positive control. Then, 50 μM Blebbistatin was applied to both the upper and lower chamber. Cells were allowed to migrate for 16 h at 37 °C. Migrated cells collected from the bottom wells were quantified by hemocytometry.

For two-dimensional (2D) migration assay, cells were seeded in 96-well plates coated with fibronectin. Time-lapse images were acquired on a JuLi Br Live Cell Analyzer (JULI-BR04, NanoEnTek, Inc., Seoul, Korea). For speed quantification and tracking, the images were acquired every 3 min for 16 h at 37 °C after the addition of blebbistatin. The instantaneous speed represented the instantaneous distance divided by time. The mean cell speed was the mean of all the instantaneous speeds of a cell. Image J software (National Institutes of Health, Bethesda, MD, USA) was used for image analysis.

### 4.12. Mice and Tumor Experiments

Male NOD-SCID mice (eight weeks old) were purchased from Orient Bio Inc. (Seongnam, Korea) and housed in a specific pathogen-free animal facility at CHA University (Seongnam, Korea). All experiments were approved by the Institutional Animal Care and Use Committee of CHA University (IACUC 190033, approval date: 18 February 2019). NOD-SCID mice were subcutaneously injected in the right flank with 2 × 10^6^ HL-60 cells. When tumors reached >5 mm in diameter, mice were treated with intraperitoneal injections of vehicle or blebbistatin (2 mg/kg, Cayman Chemical, Ann Arbor, MI, USA) every three days. Tumor volumes were assessed with a digital caliper and calculated using the formula 1/2 × A × B^2^, where A is the longest diameter and B is its perpendicular diameter. For survival analysis, the mice were euthanized when the tumor volume exceeded 1500 mm^3^ or when the mice became moribund.

### 4.13. Histologic Analysis

For immunofluorescence staining, the tumor samples were fixed, processed, and cryosectioned as previously described [54]. Tumor sections were incubated at room temperature for 3 h with the following antibodies: anti-CD31, anti-Caspase-3, and anti-Ki-67. The sections were washed triple times and incubated with the following secondary antibodies: Cy3-conjugated anti-hamster IgG (Jackson ImmunoResearch, Grove, PA, USA) and FITC-conjugated anti-rabbit IgG (Jackson ImmunoResearch). Finally, the slides were analyzed using an LSM 880 confocal microscope (Carl Zeiss, Oberkochen, Germany). ImageJ software (National Institutes of Health, Bethesda, MD, USA) was used for density measurements of apoptotic area and proliferative area.

### 4.14. Statistical Analyses

All experiments were performed in triplicate at a minimum. Data are presented as mean ± SEM or median ± min/max, unless otherwise specified. A statistically significant difference between the two groups was evaluated by two-tailed unpaired Student’s *t* tests. Multiple comparisons were performed using one-way analysis of variance (ANOVA) with Bonferroni correction. Significant differences between Kaplan–Meier survival curves were analyzed using the log-rank test. Statistical analyses were performed using Microsoft Excel and GraphPad Prism 8.0 software (GraphPad Software, Inc., La Jolla, CA, USA).

## Supplementary Materials

Supplementary materials can be found at https://www.mdpi.com/1422-0067/21/10/3460/s1. Figure S1. The localization of NMIIB and BloodSpot analysis of NMII isoforms mRNA expression in human AML across cytogenetic subtypes relative to normal HSC and progenitor controls; Figure S2. Cell viability in myosin phosphorylation-deficient HL-60 cells; Figure S3. Perturbation of actomyosin contractility effects mobilization of HL-60 cells; Figure S4. The changes in oncogene expression in human leukemia cells after blebbistatin treatment; Table S1. Details of the primary AML patient samples studied.
