# Supplementary material for: Targeting Actomyosin Contractility Suppresses Malignant Phenotypes of Acute Myeloid Leukemia Cells
Source: Int J Mol Sci. 2020 May 14;21(10):3460. doi: 10.3390/ijms21103460 (PMC7279019; doi:10.3390/ijms21103460)
Supplement: Supplementary file 1 [file ijms-21-03460-s001.pdf]

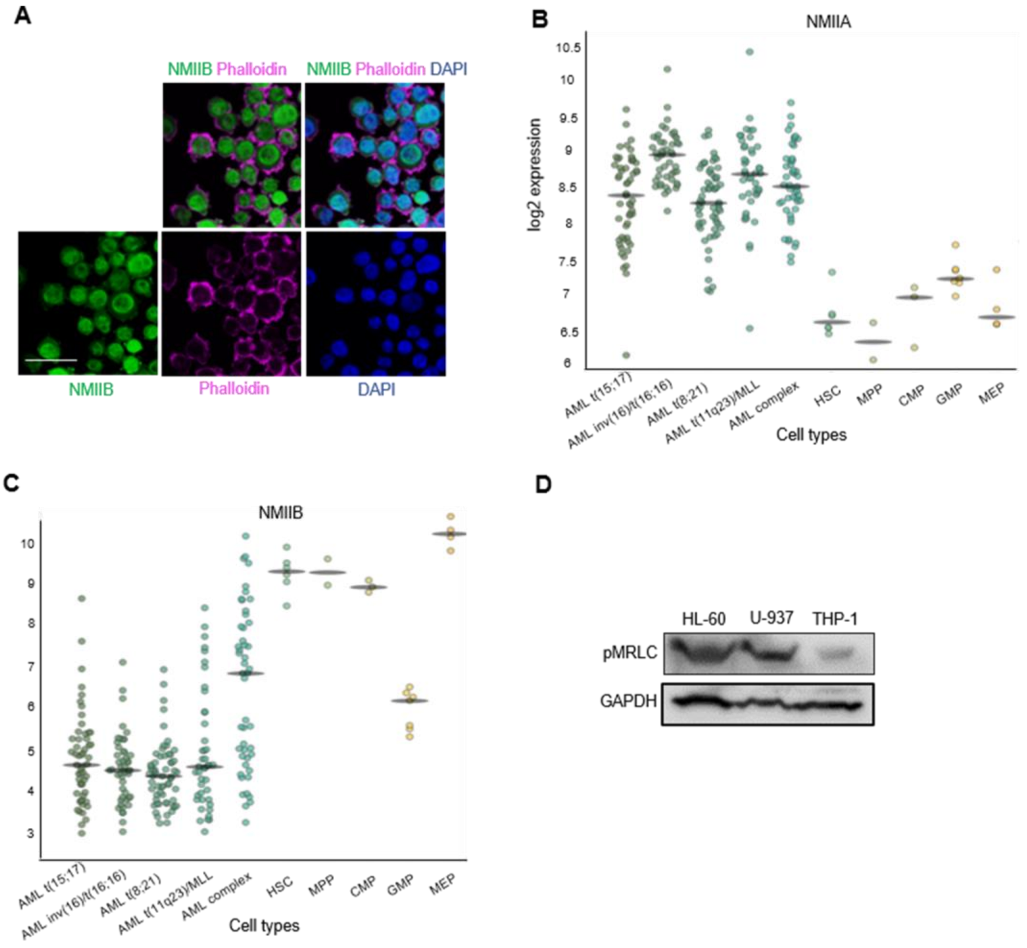


Figure S1. The localization of NMIIB and BloodSpot analysis of NMII isoforms mRNA expression in human AML across cytogenetic subtypes relative to normal HSC and progenitor controls. (A) The localization of NMIIB (green) and their spatial relationship with phalloidin (magenta) and DAPI (blue) in AML cell line HL-60. Scale bar: 50 μm. (B) NMIIA was elevated in human AML subtypes, including t(15;17), inv(16), t(8;21), t(11q23), and complex karyotype when compared with HSC, multipotential progenitor (MPP), common myeloid progenitor (CMP), granulocyte monocyte progenitor (GMP), and megakaryocyte-erythroid progenitor (MEP) subsets. (C) NMIIB was not elevated in human AML. BloodSpot is a curated database of publicly available gene expression datasets. (D) Immunoblotting of pMRLC expression in AML cell lines (HL-60, U-937 and THP-1).

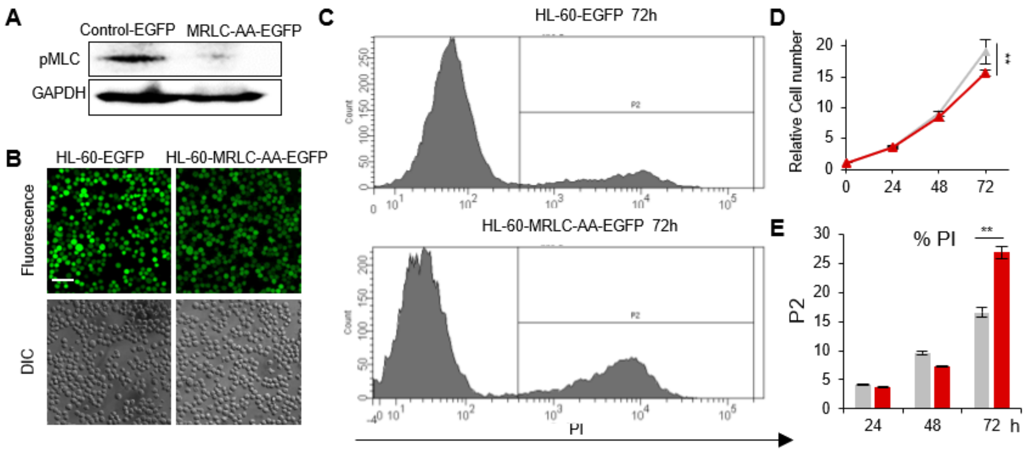


Figure S2. Cell viability in myosin phosphorylation-deficient HL-60 cells. (A) Myosin phosphorylation deficiency in EGFP and MRLC-AA-EGFP overexpressing HL-60 cells was checked by immunoblotting. (B) Immunofluorescence images of transduction efficiency in EGFP and MRLC-AA-EGFP overexpressing HL-60 cells. (C) The level (P2) of cell death in EGFP and MRLC-AA-EGFP overexpressing HL-60 cells at 72 h. (D) Comparison of viable cells of EGFP and MRLC-AA-EGFP overexpressing HL-60 cells in a time-dependent manner (n = 3). Data are represented as mean ± SEM. (E) Quantification of death signals of EGFP and MRLC-AA-EGFP overexpressing HL-60 cells in a time-dependent manner (n = 3). Data are shown as mean ± SEM. Scale bar: 50 μm (B). ***P* < 0.01.

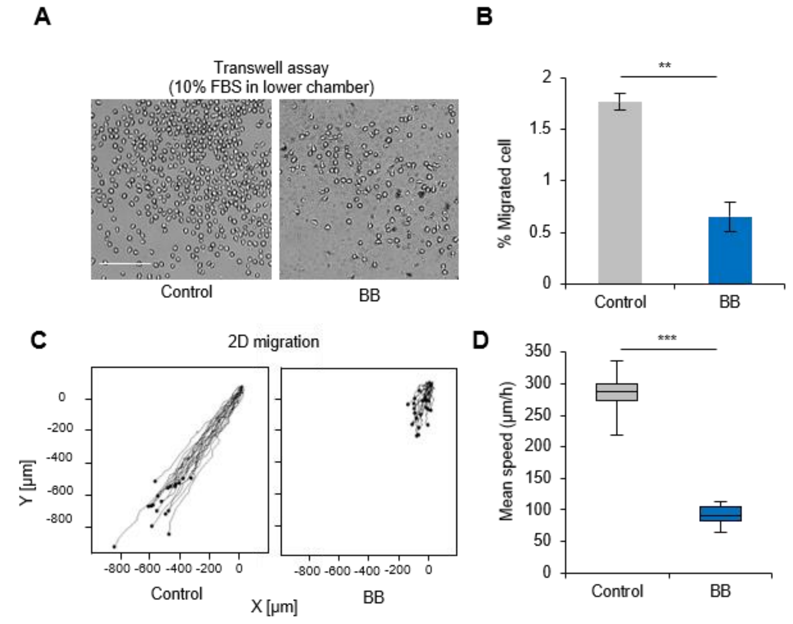


Figure S3. Perturbation of actomyosin contractility effects mobilization of HL-60 cells. (A) Representative images of migrated HL-60 cells were captured after 16 hours migration. (B) The percentage of migrated HL-60 cells upon blebbistatin treatment in a Transwell assay (n = 3). Data are presented as mean ± SEM. (C) Migration path of HL-60 cells on a two-dimensional (2D) migration platform. (D) Quantification of the mean speed changes of HL-60 cells after blebbistatin treatment (n = 30 per group). Data are represented as median ± max/min. Scale bar: 100 µm (A). **$p < 0.01$. ***$p < 0.001$.

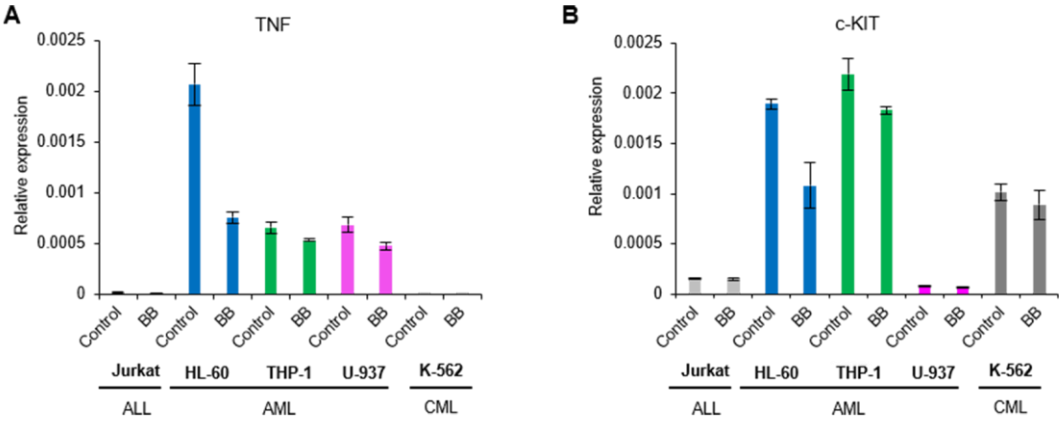


Figure 4. The changes in oncogene expression in human leukemia cells after blebbistatin treatment. (A) Blebbistatin induced TNF expression changes in human AML cell lines (n = 3). Data are represented as mean ± SEM. (B) Blebbistatin induced c-KIT expression changes in human AML cell lines (n = 3). Data are presented as mean ± SEM.

Table S1. Details of the primary AML patient samples studied.

| Sample | Patient No | CD34 blasts, % |
|---|---|---|
| AML-1 | NCC15-006 | 43.1 |
| AML-2 | NCC15-201 | 77.3 |
| AML-3 | NCC15-214 | 2.6 |
| AML-4 | NCC15-230 | 58.9 |
| AML-5 | NCC15-247 | 54.8 |
| AML-6 | NCC15-288 | 0.7 |
| AML-7 | NCC15-346 | 0.2 |
| AML-8 | NCC15-351 | 65 |
| AML-9 | NCC15-411 | 3.9 |
